# The over time development of chronic illness self-management patterns: a longitudinal qualitative study

**DOI:** 10.1186/1471-2458-13-452

**Published:** 2013-05-07

**Authors:** Åsa Audulv

**Affiliations:** 1 Department of Health Sciences, Mid Sweden University, SE-851 70, Sundsvall, Sweden

**Keywords:** Adaptation, Behavior change, Chronic disease, Development, Longitudinal, Qualitative, Self-care, Sweden

## Abstract

**Background:**

There currently exists a vast amount of literature concerning chronic illness self-management, however the developmental patterns and sustainability of self-management over time remain largely unknown. This paper aims to describe the patterns by which different chronic illness self-management behaviors develop and are maintained over time.

**Method:**

Twenty-one individuals newly diagnosed with chronic illnesses (e.g., diabetes, rheumatism, ischemic heart disease, multiple sclerosis, chronic renal disease, inflammatory bowel disease) were repeatedly interviewed over two-and-a-half years. The interviews were conducted in Sweden from 2006 to 2008. A total of 81 narrative interviews were analyzed with an interpretive description approach.

**Results:**

The participants’ self-management behaviors could be described in four different developmental patterns: consistent, episodic, on demand, and transitional. The developmental patterns were related to specific self-management behaviors. Most participants took long-term medications in a consistent pattern, whereas exercise was often performed according to an episodic pattern. Participants managed health crises (e.g., angina, pain episodes) according to an on demand pattern and everyday changes due to illness (e.g., adaptation of work and household activities) according to a transitional pattern. All of the participants used more than one self-management pattern.

**Conclusion:**

The findings show that self-management does not develop as one uniform pattern. Instead different self-management behaviors are enacted in different patterns. Therefore, it is likely that self-management activities require support strategies tailored to each behavior’s developmental pattern.

## Background

Individuals’ self-management is a crucial part of the care of people with chronic illness. In fact, most care of chronic illness is performed by individuals in their own homes [1,2]. It should be acknowledged that people are not alone in managing their chronic illness. All people are influenced – positively and negatively—by their social networks, health-care providers and society as a whole. Each of these can provide both resources for and barriers to self-management [3]. Barriers and facilitators for self-management have been described in a large body of research. For example, male sex [4], high social position [4,5], social support [6], high self-efficacy [7], and good psychological status [8] are related to performing more self-management (both in terms of frequency and types of behaviors), while belonging to an ethnic minority [8,9], being in a financially vulnerable position [4,10], co-morbidities [11], low self-efficacy [7], and demanding social obligations [6,12] are related to performing less self-management. There currently exists a vast amount of research regarding self-management barriers and facilitators; however, the developmental patterns and sustainability of self-management over time remain largely unknown. The literature that describes self-management over time mostly consists of trials to improve individuals’ self-management or small qualitative studies that describe self-management development from predominately retrospective data.

### Self-management

In this paper, self-management will be defined as the strategies a person undertakes to control disease, promote health, and live well with illness [13-15]. Behaviors to manage disease or illness-related symptoms and impairments include taking medication, seeking health-care, or managing pain. To promote health, people may exercise, eat healthy, or manage stress. Examples of behaviors to live well with illness include promoting participation, managing emotions, or managing persistent symptoms. This study focuses on self-management development patterns, defined as the patterns according to which self-management is performed over time, for example, whether a behavior is performed regularly, by increasing amounts, or episodically.

The notion of self-management evolved during the 1960s and 70s in the shadow of health-care critique (e.g., rapid increases in use of technology in health-care, power imbalance of the traditional doctor-patient relationship, and the medicalization of former nonmedical aspects of life) [2]. The idea of self-management was further influenced by changes in societal values about individuals’ responsibility for their health and ideas from the self-help movement [14]. Corbin and Strauss [16] were among the first to describe the work related to living with a chronic illness. Their model included three lines of work people undertake when living with a chronic illness: illness-related work (e.g., managing symptoms or crisis prevention, often named illness management), everyday life work (e.g., managing work or household tasks, often described as role management), and biographical work (e.g., managing emotions or identity). The first two lines of work—illness management and role management—are viewed as part of the daily process of managing a chronic condition [17]. Illness management work varies between very complex and technical work (doing dialysis at home) to quite simple tasks, such as taking a pill; often it involves both extremes. Role management work can also require planning and coordination; multiple tasks are involved in most everyday activities (e.g., self-care, household chores) and these can become difficult to maintain when people experience fatigue, pain and mobility impairments as a result of their chronic condition [17]. The third line of work, biographical work, is often not as visible as the other two lines. Biographical work is mainly, but not solely, an inner process of how individuals reformulate their identities and life goals when their chronic condition impacts their way of living. How individuals manage biographical work can impact on how they succeed in illness management and role management. Illness, role and biographical management work is always changing, depending on the stages or phases of illness, the individual’s reactions to these changes, and depending on changes in his/hers overall life situation and context. When people’s resources (e.g., equipment, finances, strength, space) are limited the lines of work can be in conflict with each other; this often requires that a person must then prioritize between undertaking illness management tasks (like following a medical regimen at home) and other valued life tasks, like attending church or volunteering in one’s community [17].

The concept of chronic illness self-management is guided by a few key principles. The first principle is that different chronic diseases often share a common set of consequences or symptoms [18]. A second principle is that persons with chronic conditions are active partners in the disease or illness management process. Health-care providers can provide some medical treatments; however the individual is the only one who can manage his or her health in a long term perspective. The third guiding principle is that people need both skills and confidence in order to be active managers of their health [19].

Self-management includes specific skills and tasks. Some self-management skills are universal for all self-management behaviors: problem solving, decision-making, resource utilization, forming of a patient/health care partnership, and taking action [19]. Other skills and tasks are related to the type of specific health condition (e.g., taking insulin requires a specific set of skills). At the same time individuals show a considerable variation in the time and devotion they put into self-management depending on life circumstances and phase of disease [15]. The term self-management can be misleading, suggesting that people manage in a vacuum. In fact, individuals always self-manage in their unique social context [20]. Peoples’ self-management support systems can consist of family members or friends, voluntary groups, and health professionals. Whereas health professionals most often focus on the disease management part of self-management the other support systems acknowledge everyday life, wellbeing and normality [21].

During the last decade many self-management programs have been developed. This movement has been particularly strong in England [22,23], Scotland [24], and Australia [25]. Research has shown people that have taken part in self-management programs have positive outcomes (e.g., self-efficacy, reduced pain, disability and improvement in depression) six to 12 months after the program, but there is little research describing longer effects [26]. There is no consensus over what constitutes a self-management program, and programs vary regarding content and delivery style [27]. Sweden (the context for this current investigation) has a short history of self-management programs. Only recently has diabetes education been available in a self-management program style. Existing self-management programs are delivered by the health-care sector and no generic self-management programs (e.g., Chronic Disease Self-Management Program) exist. Therefore people in Sweden mostly develop and maintain self-management by themselves and/or are supported by family or health-care providers.

### Self-management over time

As mentioned in the introduction, much research has investigated factors influencing self-management performance. However, the factor of time has been neglected in this research. Quantitative research that considers time has mainly focused on interventions, e.g., the length of time individuals experience positive outcomes after completion of a self-management program. Quantitative studies about how self-management develops after a person is diagnosed or how self-management behaviors fluctuate over a year are rare or even nonexistent. One exception is a study by Chriss, Sheposh, Carlson and Riegel [28]. They found that self-management levels among people with heart failure four months after discharge from hospital were best predicted by the individuals’ self-management levels at discharge.

Qualitative studies describing the process of developing self-management behaviors have predominantly focused on diabetes [29-33]. Studies do exist, however, about people with chronic renal failure [34], chronic illness in general [35] and comorbidities [36]. In these studies the process of developing self-management behaviors was described by researchers as either chronological or fluctuating. A chronological process typically begins with an individual having a fragmented understanding of his/her illness and its self-management. However, over time, the individual will reach a deeper understanding of his/her needs and integrate self-management behaviors into his/her daily life. The chronological process tends to focus on knowledge of one’s condition, learning how to self-manage one’s condition and developing expertise regarding one’s condition (e.g., [29,31,34]). In contrast, the fluctuating process relies on the argument that both life and illness experiences are ever-changing cf. [37,38]. A fluctuating process typically describes changes in individuals’ beliefs and values and the ability to overcome intrapersonal conflicts and external barriers [32,33,36]. A fluctuating process reflects phases that individuals go through in order to develop and integrate self-management into their lives (e.g., seeking effective self-management strategies, considering costs and benefits of self-management strategies, creating routines and plans of action, negotiating self-management that fits into life) as well as describes conditions which influences those phases and the individuals self-management (e.g., health beliefs, social support). However, these phases are often described as nonexclusive; individuals may shift to a phase of enacting self-management behaviors but later shift back to a phase of considering and planning for self-management, and at times, individuals can engage in several phases at the same time (e.g., managing to change their diet but being unable to stop smoking) [35].

The existing research about how self-management is developed and enacted over time has typically relied on participants with a single diagnosis and used retrospective approaches. This paper seeks to explore the notion of how different self-management behaviors are developed and engaged in overtime and to investigate the types of self-management behaviors that follow similar developmental patterns. If self-management behaviors develop and change according to different patterns, then health professionals may need to tailor self-management support strategies to these patterns of behavioral development.

### Aim

This paper aims to describe the different chronic illness self-management behavior patterns that individuals develop and maintain over time.

## Method

### Interpretive description

Interpretive description is a qualitative approach designed to answer clinical questions rather than to develop theory, understand lived experience or culture [39,40]. While some classic qualitative methods, such as writing memos or strategic sampling, are used in an interpretive description approach, the theoretical foundation of interpretive description is within a traditional naturalistic inquiry perspective rather than from a grounded theory, phenomenology or ethnographic perspective [39]. From an interpretive description perspective, for example, the research should: (a) be conducted in a naturalistic context, (b) recognize that the researcher and the research are inseparable, (c) reflect over time and context, and (d) acknowledge the existence of socially constructed elements in human experience [39]. The reasons why an interpretive description approach was chosen for this study were that the approach was suitable for analyzing large data materials, longitudinal data and the goal of the approach was to develop knowledge applicable to clinical practice cf. [39].

### Data collection

Specialist nurses at an outpatient clinic in the north of Sweden selected people who were newly referred to the clinic and fulfilled the inclusion criteria. The outpatient clinic was placed in a middle sized Swedish city (about 100 000 inhabitants) and the clinic specialized in medical conditions (i.e., rheumatology, cardiology, neurology, endocrinology). The inclusion criteria were; being older than 18 years old, Swedish-speaking and diagnosed with a chronic disease within five months of the first interview. The intention was to include individuals with diverse diagnoses in order to obtain variation in the participants’ self-management descriptions, as well as different illness trajectories. The recruitment took place over a seven-month period from March 2006. The selected individuals received an information letter inviting them to participate in the study. A few days after receiving the letter, potential participants were contacted by phone and were offered additional information. At total, 32 individuals received an invitation and 22 agreed to participate (one woman dropped out after the first interview and was excluded). The participants gave verbal informed consent before the interview. Both the letter and the additional information included a guarantee of confidentiality, that health-care service would not be affected by participation and that the participants had the right to decline at any time without giving a reason. The purpose of the study was described as investigating individuals’ adjustments, management and healthy living when diagnosed with a long-term condition.

At the first individual face-to-face interview, the participants had been diagnosed between one and five months earlier. The second interview was conducted six months later (6–11 months after diagnosis), the third was conducted one year later (12–17 months after diagnosis), and the fourth and final interview was conducted 2.5 years after the first interview (30–35 months after diagnosis). The intention was to conduct the first interview as soon as possible after the participants received their diagnosis. However, because some of the eligible participants were admitted to the outpatient clinic weeks or even months after they received their diagnosis, a few participants were interviewed three or four months after their diagnosis. As noted, one participant did not wish to participate in the second interview. That case was excluded from the analysis. Three participants took part in three out of the four interviews and these cases were included in the study; one of them chose not to take part in the 12 month interview (because of personal reasons) but participated in an interview 30 months after receiving his diagnosis. Two people could not be reached for the 30 month interview.

The participants chose the location for their interviews (at their homes, places of work, or in a private room at the university). The first interview began with the question ‘What was it that made you seek care?’ Thereafter, the participants were encouraged to tell their illness stories. The interviewer used probing questions to encourage the participants to talk and to develop their stories. Questions about self-management were brought up in every interview (e.g., “How do you manage your illness? What do you do to manage your symptoms?”). The subsequent interviews all began with “How are you, and how have you been doing since we last met?” In preparation for the later interviews, a recording of the previous interview was listened to, and follow-up questions were noted (e.g., “At our last meeting, you were talking about your plan to stop smoking. How has that evolved?”). In cases where participants mentioned few self-management behaviors the interviewer probed for examples by referring to behaviors mentioned by other participants (e.g., So how do you manage stress?). The interview guide used is published elsewhere [35]. Most interviews were between 40 minutes and an hour long; the shortest interviews were 20 minutes and the longest approximately two hours. The longer interviews tended to cover more context than the shorter interviews, and participants in longer interviews tended to describe severer symptoms, more self-management behaviors and greater impact of illness. The interviews were recorded and transcribed verbatim. All names used in the results are pseudonyms. The study was approved by the Regional Ethical Review Board in Umeå (No. 05-164M), Sweden.

### Participants

The sample consisted of 21 participants who were interviewed on three to four occasions over a two-and-a-half year period (e.g., 2006–2008). In total, the analysis included 81 interviews.

Twelve women and nine men took part in this study. The participants were recruited because they had received a new diagnosis of one of the following conditions: ischemic heart disease (n = 4), rheumatic disease (n = 5), chronic renal disease (n = 3), inflammatory bowel disease (n = 3), multiple sclerosis (MS) (n = 2) and diabetes (n = 4). Eight participants had chronic conditions prior to their new diagnosis. Such conditions included asthma, high blood pressure, breast cancer, and fibromyalgia. At the time of the first interview, the participants’ ages ranged between 20 and 74 years old (median 47). Fifteen of the participants were married or cohabitating. Fourteen participants were employed, two were unemployed, and five were retired. At the final interview, one participant received a disability pension, one retired early, and four reduced their working hours due to illness.

### Analysis

Transcripts were analyzed using an interpretive description analysis. Interpretive description analysis typically begins with getting a broad understanding of the entire data set, and later, the analysis becomes more focused and specified [39]. At the beginning of this analysis, all sentences that described self-management were identified and allocated into categories of different types of self-management behavior. Examples of the categories include: symptom management, navigating the health-care system, stress management, and adaptation of work.

The next suggested phase in interpretive description is to move from viewing the data in pieces to identify patterns [39]. Each participant’s set of interviews was condensed into a matrix, in which all of his/her self-management behaviors were described. Using this procedure, any changes in self-management behaviors were illuminated. Differing self-management behaviors were then arranged together, to determine how participants changed according to specific behaviors. During this stage, it was noted that the different self-management behaviors followed different patterns.

An important part of interpretive description analysis is illuminating and exploring relationships within the data in order to determine how different patterns differ from or relate to each other [39]. In this study the patterns were compared with each other. The analysis questions were, for example: “What are the characteristics of the pattern of diet over time?” From this, four different self-management patterns were identified. The next and last step was to explore and conceptualize the self-management patterns. According to interpretive description the conceptualization of findings is the final step of the analysis where patterns are described both in depth and in relation to each other [39].

A number of steps were also taken to ensure trustworthiness. For example, memos were used to track the analysis process. Furthermore, the analysis was repeatedly discussed with other researchers to identify weaknesses and negative cases, get new ideas of how to name the categories and conceptualize and interpret the findings.

## Results

During the two and a half years the participants were interviewed, they exhibited different patterns of how their various self-management behaviors were developed or maintained. In total, four patterns of self-management were found: consistent, episodic, on demand, and transitional. Some self-management behaviors were described as one self-management pattern. For example, when participants described how they managed to continue working with paid employment despite their condition, it was described as a transitional self-management pattern; Participants changed their attitudes and approaches to work and learned to perform work-related tasks in new ways. Other self-management behaviors were described in two different patterns. For example six participants described that they exercised regularly (a consistent self-management pattern) during the interview period and 13 participants described how their exercise was more sporadic (an episodic self-management pattern). However, a single person often showed all or several of the self-management patterns (see Table 1). No participant was found to use only one self-management pattern.

**Table 1:** An example of an individual’s self-management behavioral development in the various self-management patterns

**Type of pattern**	**A few weeks after diagnosis**	**Six months after diagnosis**	**12 months after diagnosis**	**30 months after diagnosis**
**Type of self-management behavior**				
**A consistent self-management pattern**				
Long-term medication	Anne takes anti-rheumatic drugs. She is concerned about the risks of side effects.	Anne takes anti-rheumatic drugs.	Anne takes anti-rheumatic drugs and has no side effects, but she is concerned about the risks and has decided to ask her physician about stopping the medication.	Anne is still taking her anti-rheumatic drugs. She has discussed the side effects with her physician and has concluded that her treatment will be life-long.
**An on demand self-management pattern**				
Managing painful periods	Anne describes that she has to listen more carefully to her body. She uses pain medication during periods of severe pain.	Anne is less active during painful periods. She does not want to use pain medication but does occasionally when the pain becomes extreme.	When she has pain, Anne is more aware of her activities and pace, and she prioritizes more.	When she encounters severe pain, Anne describes “enduring” as her only strategy. She uses pain medication occasionally, for example, to sleep.
Seeking information	Anne read quite a lot about RA on the Internet after she received her diagnosis.	Anne reads about RA when she comes across an article, but she does not want the illness to take up too much of her life.		Anne says she knows quite a lot about RA, and she does not need to know everything.
**An episodic self-management pattern**				
Exercise	Anne claims that exercise is more important now. She has created routines for regular swimming and walking exercises.	Anne has swum less during the last month because of more obligations at work.	Anne has mostly walked during the summer holiday. She has had a break in her swimming exercise, and gym exercise has been difficult because of increased pain.	Anne performs regular exercise, and she adapts the type of exercise to her current health status. In more painful periods, she is unable to take walks, but she can swim.
**A transitional self-management pattern**				
Managing leisure activities, work and using self talk strategies	Anne has begun to plan for a different life. She perceives an increased need for recovery and rest. Anne has tried to change her attitudes and allow herself to slow down at home and at work. She has given up some career plans and leisure activities. She avoids thinking of the future, when she might get worse.	Anne continues to change her priorities; she saves more for her retirement but spends more on holidays. She uses self talk strategies to cope with performing less, both at work and at home. She can perform activities that lead to pain if they are valuable to her. At times, Anne allows herself to grieve over her situation.	Anne describes her health as fairly good, and she wants to focus on her abilities, not her losses. She has developed strategies to take short rests in her everyday life. Anne has taken an active part in decreasing her workload, and she has adapted some activities.	Anne wants to continue living a good and healthy life. To do so, she lives in a more scheduled, less spontaneous manner and gets more rest. She has slowed down her life and prioritizes among her activities. Anne claims that she also needs to be able to ignore her RA. She describes her transition process as a developmental process that is influenced by aging and maturation, as well as her RA.

Diagnosis: Rheumatoid arthritis (RA).

In total 15 participants described the need for life transitional changes; including most participants with rheumatism, MS, and diabetes, two participants with ischemic heart disease and one participant with renal disease. The seven participants that did not describe transitional changes (they lived with ischemic heart disease, inflammatory bowel syndrome, and renal disease) had fewer symptoms and stated that their disease had little impact on their everyday life. However, it should be noted that all participants were rather early in their illness experiences having had their disease for approximately three years at the final interview and some had not developed severe symptoms.

### A consistent versus episodic self-management pattern

A consistent self-management pattern was represented when participants continued with self-management behaviors without major changes during the entire interview period. The most common consistent self-management behavior was taking long term medications (e.g., anti-rheumatic drugs, blood pressure medications, insulin, multiple sclerosis-modifying treatments). Many participants were consistent with eating healthy and a few participants were consistent with their exercise (see Table 2). Allan described how he anticipated taking medication for the rest of his life after having had a conversation with his physician: “You will have to take medication [said my physician], you will have to take it for the rest of your life as well. I have adapted myself to that, and it doesn’t feel like any sacrifice” (ischemic heart disease, 6 months after diagnosis). It was important to develop individualized routines in order to maintain a consistent self-management pattern. Philip described how he decided to buy a dog to be more consistent with his exercise:

Even though I take walks because I must do it… must do it and because it is good for the diabetes. So I thought that a dog would help. Every day, it would be a couple of times, some longer and some shorter [walks]. It would help with the disease as well. (Diabetes, 30 months after diagnosis)

**Table 2:** Description of self-management behaviors that were performed with either a consistent or episodic self-management pattern

**Type of Self-management behavior, number of participants describing behavior**	**Number of participants with a consistent pattern (diagnoses)**	**Number of participants with an episodic pattern (diagnoses)**	**Other**
	**Description**	**Description**	
**Long-term medication**			
N = 21	N = 17	N = 1	Two participants stopped taking medication after consulting with their physician. (rheum, MS)
	(all diagnoses)	(IBD)	One participant stopped taking her medication by herself. (rheum)
	Participants continued to take medication as prescribed.	One participant stopped with prescribed medication which he did not find effective. Started taking another medication and later took the first medication again.	
**Exercise**			
N = 21	N = 6	N = 13	
	(CRD, diab, IHD)	(diab, IHD, IBD, rheum, MS)	
	Walks were the most common type of exercise. Five participants exercised regularly before being diagnosed.	Participants described having the intention to exercise but maintained the behavior in periods.	Two participants decided to stop exercise. (CRD)
**Healthy diet**			
N = 16	N = 11	N = 5	
(all diagnoses)	(IBD, diab, IHD, CRD, rheum)	(diab, CRD, rheum, IBD)	
	Participants described being consistent with eating healthy.	Participants described eating healthy as problematic; especially regarding having regular meals and some wanted to know more about how different foods affected their health.	
**Smoking (and snuff) cessation**			
N = 3	N = 1	N = 1	
(diabetes, ischemic heart disease, rheumatism)	(IHD)	(diab)	
	One participant stopped smoking at diagnosis, and described that she smoked one time during the study period.	One participant stopped using snuff, later started and prepared to stop again.	One participant described that she wanted to stop smoking but she did not try during the study period. (rheum)
**Monitoring**			
N = 5	N = 1	N = 3	
	(diab)	(diab)	
	One participant continued to measure blood glucoses at least once a day.	At the beginning of the study participants measured blood glucoses several times a day, over time they limited their measuring. Participants still measured blood glucose levels several times a week, but they did not take series or before or after meal tests.	

Diagnoses: Inflammatory bowel disease (IBD), Multiple sclerosis (MS), Rheumatism (rheum), chronic renal disease (CRD), Diabetes (diab), ischemic heart disease (IHD).

In contrast, self-management behaviors performed in an episodic self-management pattern were intended to be consistent but ended up being maintained in periods of action and non-action. Examples of behaviors that often were carried out in an episodic pattern were exercise, eating healthy, smoking cessation, and monitoring blood glucose values. The participants’ goal when expressing these behaviors was that they should be performed according to a consistent self-management pattern, however during the study period they were unsuccessful in achieving this goal. Reasons why self-management behaviors ended up being performed in an episodic self-management pattern were that participants found it difficult to perform behaviors regularly over a long period of time. The participants needed a routine and motivation to be able to continue with some self-management behaviors. For example, the participants experienced difficulties in maintaining their exercise regimens when their routines were challenged. Tom described the reasons to why he could not maintain his regular exercise:

So it’s been a while [since I exercised]. And then you get a cold, or it is a holiday, and it disappears, and you lose focus and… well, it is easy to come out of it [the exercise]. (Ischemic heart disease, 12 months after diagnosis).

The participants who were most likely to sustain healthy lifestyles during the study period were the ones who had exercised or kept to a healthy diet before, as they had already developed routines that worked to support their self-management. Vicky described how she and her late husband had changed their diet together after he was diagnosed with diabetes and that she maintained the healthy diet after her husband had passed away:

I try to eat good healthy food and that I had learned because umm [my husband] was a diabetic for 12 years. So I have learned to eat unsweetened soup and more vegetables, decrease the fat, no cream sauces and I have continued with that. (Ischemic heart disease, 6 months after diagnosis)

For other participants lifestyle changes were harder to maintain in their daily lives. For example exercise could be difficult for participants with chronic or episodic pain and/or fatigue. Some participants with rheumatism described how they adapted their exercise, depending on current symptoms. For example Anne alternated between walks, gym exercise, and swimming depending on her levels of pain: “If I have pain, then I notice that the only exercise I can do is swimming […]. I do not go out and take walks if I have a whole lot of pain in my feet” (rheumatism, 30 months after diagnosis). Other people with chronic pain struggled during the two and a half years to find an exercise they could perform despite their pain. For Emma, exercise had been an important part of her life before she developed rheumatism but after her diagnosis it was difficult for her to maintain fitness: “Because I can’t move the way [I used to]. I can’t go on speedy walks or bike or so. I don’t use the weighting-machine, but can feel it [increased weight] on the clothes” (12 months after diagnosis).

### An on demand self-management pattern

An on demand self-management pattern existed when participants performed certain self-management behaviors occasionally to try to control acute symptoms or short-term events, such as a health crisis. For example, they used strategies on demand: to manage angina, or during active periods of inflammatory bowel disease, or periods of increased pain, or hypoglycemic events (see Table 3). The on demand pattern was only intended for events with a short time-span, often to control symptoms and limit the effects of diseases. This is in contrast to the episodic self-management pattern that was used for self-management to improve long term health and intended to be maintained over time. Christine had ulcerative colitis and described how she managed an illness episode by limiting enjoyable but stressful activities for a few weeks. Among other things, she took a break from her choir practice and cancelled her participation in a family celebration. She did this in order to decrease stress that impacted on her symptoms. At the time of the interview she was just about to start taking up her activities again:

Then, in the spring it became too much for me. (…) Then, we were going [to my grandchild’s confirmation], and there were many things, and then, I did not feel well. And then, I stopped everything; I didn’t even go to the concert — nothing, I had to. However, now I’m fine again; my choir practice begins this evening. (6 months after diagnosis)

**Table 3:** Self-management behaviors described in an on demand pattern

**Type of self-management behavior, number of participants describing behavior(diagnoses)**	**Description**
**Manage acute symptoms**	Type of symptoms:
N = 14	Hypoglycemic events (n = 4),
(diab, IBD, IHD, rheum)	Heart symptoms (n = 5), diarrhea and pain (n = 2),pain (n = 3)
**On demand medication**	Type of medication: pain medication, cortison, anti-depressive medication
N = 7	
(rheum, IBD, MS)	
**Information seeking**	Initially, 10 participants described information seeking.
N = 14	During subsequent interviews, 9 participants described sporadic information seeking about their disease.
(all diagnoses)	
**Navigating health-care**	Type of contact: follow ups, sought advice and tests when symptoms worsened, advocated to receive treatment or consultations.
N = 14	
(IBD, rheum, IHD, MS)	

Diagnoses: Inflammatory bowel disease (IBD), Multiple sclerosis (MS), Rheumatism (rheum), chronic renal disease (CRD), Diabetes (diab), ischemic heart disease (IHD).

The participants were active in searching for health-care or information in situations during which their illnesses were in focus, for example, when they first received their diagnoses or when their symptoms worsened. Emma first tried to manage her rheumatic pain on her own, but when it increased in intensity she advocated for a follow up consultation to get help to manage the pain:

Then, it was just getting worse. I had extreme back pain, very hard. (…) And then, it hadn’t really become any better, so I began seeking physicians again, because I wondered: shouldn’t there be a follow-up on my medication or something? Yeah, and then I wanted a plan for what would happen thereafter. (6 months after diagnosis).

The participants developed their strategies to deal with health crises over time. A strategy presupposed some knowledge of underlying disease mechanisms, body listening and knowledge of self-management strategies. Mike, who had been diagnosed with diabetes six months earlier, stated that his blood-glucose levels fluctuated and were often very low. To manage these low glycemic events, he ate sweets and reduced his insulin doses:

Mike: No… like it ought not be… I should not be as low [in blood glucose level] as I am.Interviewer: What do you do about it?Mike: Eat sweets (short laugh). I skip the insulin, I do. I take very little… (6 months after diagnosis).

Half a year later, Mike had developed a better understanding of the actions and situations that preceded low glucose events, and he often managed to avoid having low blood glucose.

Yes, well it is, it is mostly when I’m careless, or I’m somewhat careless with the food, or I don’t always have time to eat when I should eat. So it’s, well, mostly that I go low because I don’t have time to eat, really. (12 months after diagnosis)

### A transitional self-management pattern

A transitional self-management process was marked by a change in life focus, the identification of new needs, acceptance of the situation, changing of values, acquiring of knowledge, and strategy development. For example, in their first interviews, some of the participants described how they were searching for practical strategies to limit pain or simple rules that would help them to balance their blood glucose levels. In later interviews, they had changed their focuses on self-management and had tried to find ways to accept and live with pain or to change their life structures. The participants also stated that they had begun appreciating other things in life and prioritizing themselves more. Both Kathy and Philip describe how they have learned to live with their illness:

You are able to feel well despite having pain every day. You do not have as much pain every day; some days you experience yourself almost as free of pain, despite that you aren’t. It is weird that, you have heard others say, you can learn to live with pain and that you can. It’s nothing that’s overwhelming at the end. (Kathy, rheumatism and fibromyalgia, 30 months after diagnosis)

Most of all, my family says that I have become humbler. I put more value in my close friends and my family, not putting work first. It has become third, which feels good also. (Philip, diabetes, 30 months after diagnosis)

The goal of self-management within a transitional pattern was to be able to continue with activities, live a good life despite illness, and slow down to prevent the illness from becoming worse. To slow down, the participants used many self-management strategies; for example they needed to adapt working situations, leisure activities and household activities to manage their symptoms, such as pain or fatigue (see Table 4). Successful management of fatigue and pain took the form, for example, of prioritizing, pacing, adapting activities or replacing former interests. Margret described how she changed the way she planned her everyday life: “You can’t go at the same speed as you did before; you must take it easier and plan another way. Not so you book too much in the same week; instead, that you take it a little slower”. (MS, 30 months after diagnosis)

**Table 4:** Self-management behaviors in a transitional self-management pattern

**Type of self-management behavior, number of participants describing behavior (diagnoses)**	**Description**
**Pain management**	In the beginning, participants described strategies to try to control or limit pain (e.g., warm baths, pain medication). Later, they used strategies to live with pain including pacing, prioritizing and self talk strategies.
N = 7	
(rheum, MS, IBD)	
**Fatigue management**	In the first interviews the participants did not know how to handle “tiredness”. Over time, the participants integrated the following strategies in their daily life; pacing, prioritizing, planning for rests, and regular sleeping hours.
N = 8	
(rheum, IHD, CRD, MS)	
**Managing household activities**	Participants changed how they conducted everyday household activities. They used pacing and prioritizing, asked for help, bought services, or adapted their ways of doing things.
N = 12	
(Rheum, IHD, IBD, MS, CRD)	
**Manage work**	The participants started with smaller changes; for example changing working tasks or tried to limit stress at work. Later they changed their working environment (e.g., a quite room, an ergonomic chair) in collaboration with employers, changed their attitude to working (e.g., more relaxed and less competitive) and some limited their working hours. A few participants changed their place of work or stopped working.
N = 13	
(rheum, diab, IHD, CRD, MS)	

Diagnoses: Inflammatory bowel disease (IBD), Multiple sclerosis (MS), Rheumatism (rheum), chronic renal disease (CRD), Diabetes (diab), ischemic heart disease (IHD).

A transitional self-management process could also be emotionally painful. Some participants described how they grieved an identity loss or trading off activities. They adapted to living with illness, which resulted in limited possibilities for being spontaneous and a loss of appreciated interests. John, who had several comorbidities, had not been able to replace his former interests in building and renovation. Meanwhile, Carol who had had a difficult period with pain, hardly leaving her home, had found strategies to manage the pain and planned to take up her studies:

I have finished with about everything. Earlier, you could work some here in your neighborhood, but now I don’t care anymore. (John, chronic kidney failure, ischemic heart disease, lung disease, a few weeks after being diagnosed with chronic kidney failure)

That’s just about pulling yourself up. I don’t know really how you should do it; instead, you get to choose. Either you lie there and feel bad or you think; now, I shall manage this. Then, you just do it. (Carol, rheumatism, 30 months after diagnosis)

Some of the participants were still searching for ways to live after having had their illnesses for almost three years. They realized the need for change but lacked the strategies and support to change their lives. Emma described how she had been unsuccessful in seeking employment that would suit her needs better; limited support and many obligations being a single parent also impacted her abilities to self-manage:

Now I’m in some sort of a [phase]… what should I do now? I’m this ill. I will not be able to work more [than part-time]. I will not get well. What should I do now? Somewhat blue… Searching for alternatives, gloomy, no man’s land… Waiting at the opening of some door… Will it work? Will it work out well for me? (rheumatism, 30 months after diagnosis)

### An on demand versus a transitional self-management pattern

Some self-management behaviors were described with an on demand pattern by certain participants and with a transitional pattern by others; examples included stress-management or coping strategies, self-talk strategies, or managing leisure activities (see Table 5). When these self-management strategies were described as on demand, the participants understood the problem as short term, symptoms as temporary and stated their illnesses as a whole had minor impacts on their lives. For example, some participants described employing mental strategies on demand when they were recently diagnosed or when they experienced exacerbations. Kevin described how he was afraid of being alone and had sleeping problems a few days after his heart attack. To cope with his fears, Kevin’s grown up children supported him and he used sleeping pills for a few days afterwards. Later in the same interview, Kevin described that he no longer thought much about the event:

So… that was an ordeal. You pondered it a lot, how it would be… because I couldn’t be alone. I noticed [that] when I got home. It was a damn problem. I was lucky that I have a daughter living close who supported this, so she was here. She was probably here, well, six… seven days. Even the son was up here, so he was [here] some days as well. Then you feel a little safer, I needed that then. Even though they were here I had to take those pills because I slept so damn bad. I could fall asleep, then I slept ten minutes and then I woke up again, then couldn’t sleep… it never became restful sleep. I had to take those [sleeping pills] because at the same time the body needed rest, I had to take those. (A few weeks after diagnosis)

Now I don’t think much upon the heart attack. And you can’t contemplate too much either, because I could have a new heart attack tomorrow, obviously. But I can’t do much about that. […] So I got over it, I believe I feel quite well both physically and mentally. (A few weeks after diagnosis)

**Table 5:** Self-management behaviors that could either follow an on demand or transitional pattern

**Type of self-management behavior, number of participants describing the behavior**	**Number of participants with an on demand pattern**	**Number of participants with a transitional pattern**
	**Description**	**Description**
**Stress-management**		
N = 9	N = 4	N = 5
	(IBD, IHD, CRD)	(rheum, IHD, diab, MS)
	Participants described how they managed stress when they were in a stressful period, e.g., when their workload caused symptoms.	Participants realized a need to manage and limit stress in their everyday life. They prioritized, planned and could for example start buying home cleaning services.
**Managing leisure activities**		
N = 14	N = 5	N = 9
	(IBD, diab, IHD)	(all diagnoses)
	Participants did not engage in activities during periods when they had more health problems.	The participants changed the way they performed leisure activities; they evaluated their activities, took up previous activities and stopped doing some.
**Self-talking strategies**		
N = 18	N = 6	N = 10
	(CRD, IBD, IHD, MS)	(rheum, diab, IHD, MS)
Two participants described self-talking strategies too briefly to be classified.	Participants used self-talking strategies only in situations when their disease became problematic (e.g., when the disease had symptoms or a participant was hospitalized).	Participants used self-talking strategies in everyday life as a way to enhance health and participation.

Diagnoses: Inflammatory bowel disease (IBD), Multiple sclerosis (MS), Rheumatism (rheum), chronic renal disease (CRD), Diabetes (diab), ischemic heart disease (IHD).

In contrast, the participants who described the need for transitional changes had to deal with more persistent negative feelings in relation to severe and long-lasting symptoms, decreased ability, disability, and/or illnesses that affected their work, leisure activities and social lives. Peter described how, on the one hand, he started to get used to diabetes management but, on the other hand, found his situation frustrating and restrained his social participation:

Now it has passed so long [time], at the beginning it was so clear regarding how much you changed your lifestyle. Now it is more like… now you begin to be more used to it, [you] are a little more withdrawn. Your mood is affected also, you are going to do something and you can’t do everything, then it’s not as fun anymore. You go to the pub and not… yeah… can’t follow the guys in the way you would want to. You go visit a friend and you do bring your syringes, are going to have lunch in town, so you eat your lunch and then some other things happens, maybe you can’t accompany [them] because you haven’t had your snack or maybe not [brought] your dinner insulin or whatever, then it’s just to go home. (Peter, diabetes, 6 months after diagnosis)

Some participants would try to manage stress with strategies focused on managing immediate problems (e.g., trying to limit the influence of stress for the next few days or weeks). For example, Kevin described how his workplace underwent major changes and, as a result, everyone worked overtime. In the first quote Kevin described his fears of how this stress affected his health and particularly his ischemic heart disease; the other quote described how he tried to manage stress with specific strategies:

Well the stress does affect me, I have told you that before. And then [in relation to the reorganization at work] I had sleeping problems and the heart beats weirdly. It affected me. You need to think about… what I’m [potentially] causing, so I don’t get another heart attack.

Interviewer: How do you manage stress?

Kevin: Deep breaths and try to take it easy and not get stressed up. You can’t really resist and tell yourself just… it’s not so simple. But anyway try concentrate upon the most important stuff, but that… I don’t do well, really. (30 months after diagnosis)

In contrast, other participants managed stress by trying to change their personal values on performance in an effort to create a stress free life. Margret (who has MS), for example, described how stressful situations caused her body to shut down and she had changed her attitude to activities and tried to do activities in a new way to avoid getting stressed:

Well, then [in a stressful situation] that enormous tiredness reappears so I like… can’t do anything more, must go and sit down or put myself to bed. Then it is impossible to think, you can’t do anything. Or if you go to town… before [the MS] you could have a long list of things you should do, but I have stopped doing that. Now I go and then I do what I have time for and can. It’s not possible to get stressed, it’s hard to explain, it [the body] just shuts down. You have to go [to town] without preconditions. (12 months after diagnosis)

## Discussion

The aim of the paper was to describe the patterns by which different types of chronic illness self-management behaviors are developed and maintained over time. Four self-management patterns were identified: consistent, episodic, on demand, and transitional patterns. Different self-management behaviors were related to those patterns. For example, long-term medication was regularly taken in a consistent pattern, whereas chronic pain management was performed according to a transitional pattern. Behaviors related to the various patterns had different goals and time-lines. Self-management behaviors performed according to a consistent or episodic pattern were often long-term health behaviors that participants tried to incorporate in their lifestyle. Meanwhile, self-management behaviors performed on demand were behaviors that participants performed to manage an acute problem or health crisis, expecting their health to return to status quo. In contrast, self-management in a transitional pattern was a way for the participants to change their behaviors to adjust to a new way of living with a chronic illness or health condition. Chronic illness is always changing, both regarding the changes or fluctuations in symptoms and health problems and because an individual’s life is always changing [17]. This makes it extremely important to investigate self-management over time.

The self-management literature can be divided into three types of studies; 1) quantitative surveys investigating relationships between self-management and specific factors, 2) qualitative studies that either explore those relationships or self-management practices in depth and 3) interventions to support self-management behavior. In the first and second types of studies, longitudinal follow up is rare. The quantitative surveys often investigate specific self-management behavior relationships to different factors (such as self-efficacy or income level). For example, Small et al. [41] when exploring relationships between emotional expression, emotional processing and self-management divided the individuals self-management regimen into behaviors e.g., diabetes knowledge, diet, exercise, blood sugar testing, and foot care. In contrast, qualitative explorative studies often focus on self-management regimens and not on individual behaviors. For example, Riegel, Jaarsma and Strömberg [42] focused on self-management as a whole when they described a recently developed theory on the overarching factors influencing individuals’ self-care maintenance. This study is different in that it adds a description of how various self-management behaviors are enacted and maintained over time. This is previously not described with qualitative methods.

In this study taking long term medication was the most consistent performed behavior. That could depend on the fact that the participants did not find taking long-term medication difficult to perform, once they had developed routines for taking their medications. That is in contrast to large quantitative studies that describe adherence with medications to be around 50-70% [43-45]. However, quantitative studies focusing on adherence use more precise measurements and definitions for medication adherence than in this study where we relied on the participants’ own descriptions of their medication taking.

In contrast, diet and lifestyle changes were performed according to a consistent pattern by some participants and an episodic pattern by others. The results indicate that participants who managed to be consistent with their diet or exercise had developed routines and habits for those behaviors long before they got their chronic illness. Corbin and Strauss [17] suggest that management work can be made into routine, but routines are often disturbed by events or issues that disrupt the normal flow of everyday life. This seems to be one reason to why exercise was particularly difficult to maintain over time. In accordance with the literature, this study identify several barriers to exercise, like chronic pain and fatigue [36], disruption of habits [35] and lack of family support [20]. A recent review described that family members are particularly important for a person’s with diabetes exercise and diet; for example, family members can strengthen the person’s motivation for exercise or family traditions can inhibit dietary changes [20]. The participants in the study were motivated to change their lifestyles. However, they did not always possess strategies to overcome barriers, and may have needed support to take up behaviors after a “relapse”.

As described in the background, qualitative studies describing the process of developing self-management strategies and behaviors have reflected either chronological or fluctuating models [37]. However, while the patterns of self-management identified in this current study contained elements found in both chronological and fluctuating models they did not mirror any of the models as a whole. In keeping with the chronological model of developing self-management e.g. [29,31,34] the participants did increase their knowledge and became more advanced in their self-management behaviors over time. This was particularly true for self-management related to on demand or transitional patterns. However, there were participants who did not develop their self-management strategies or behaviors over time; moreover it is also possible that individuals might be experts in some of their self-management behaviors but not in others. When the participants engaged in a transitional pattern they described changes in attitudes, values and intrapersonal conflicts, which also are evident in the fluctuating model of self-management development e.g. [32,33,36].

It was interesting that some self-management behaviors (e.g., stress-management, managing leisure activities, and self-talk strategies could be performed either in a transitional or on demand self-management pattern. It is likely that the participants’ beliefs about their symptoms as an acute health crisis (e.g., strive to go back to status quo) or as lasting symptoms mirrored their approach for on demand or transitional self-management patterns. When participants understood their symptoms as chronic they needed to make substantial changes in both their everyday lives and identities. This kind of self-management is categorized as living with illness by Schulman-Green et al. [15] and includes tasks like adjusting to a “new” self, modifying one’s lifestyle to adapt to disease, seeking normalcy and making meaning of a life with illness. In contrast, participants who used an on-demand self-management pattern did not find a need to change their everyday life; instead they looked for solutions to their problems or waited for acute problems to subside. In a classic paper focusing on transition processes in women with rheumatoid arthritis, Shaul [46] described that the first stage in the women’s transition process was to become aware and seek a diagnosis and treatment. Prerequisites for this stage were when the women’s early sensations became constantly severe and a barrier in their daily activities.

### Methodological considerations

This study’s greatest advantage was its longitudinal approach, which allowed for prospective follow up on self-management development. However, some self-management actions were applied by so few participants that the patterns were not reliable. For example, only four participants tried alternative therapies, and therefore alternative therapies were excluded from the analysis. It was also somewhat problematic to obtain reliable accounts of the participants’ diets in the interviews. Most of the participants stated that they ate healthy (e.g., including vegetables, fruit and reduced fat or sugar products). However, individuals’ beliefs about, for example, amounts of food or regular meals might have differed. To obtain more precise descriptions, a diary could have been used to collect data for some self-management behaviors such as diet. Labeling self-management behaviors was also a challenging process: For example, stress management could be a category of its own or a part of fatigue management or an important aspect of managing employment and work. In this process some self-management behaviors were labeled in more than one category at the beginning and the categorization had to be reflected upon and changed several times before final decisions about categorization were made.

A few participants (n = 5) had begun with a self-management behavior in the early interviews but made a decision to stop and did not take up the behavior again during the study period (see Table 2 for details). This was seen in particular in behaviors such as long-term medication and exercise. It is possible that this is an additional self-management pattern. However the participants enacting this possible pattern were so few so the pattern could not be described in a comprehensive and credible way. Future research might answer the question if this is an additional pattern.

All of the participants lived in Sweden; consideration should be given regarding how that fact may have affected the results. Nonetheless, the overall descriptions of self-management development patterns are likely to be transferable to a wider context of people self-managing chronic illnesses. However, individuals in other countries might describe other kinds of self-management behaviors, barriers or facilitators, for example, more alternative therapies or access to self-management programs.

## Conclusions

According to the findings of this study self-management behaviors follow different patterns over time. This study identified four different patterns. All participants described using three or four different patterns depending on the type of self-management behavior. The most frequent form of continuous self-management involved adhering to medication regimens, whereas health promoting behaviors, such as exercising or healthy eating were more episodic in nature, especially for those who had not established prior patterns of healthy living behaviors. It was of interest that the work of managing the emotional consequences of living with a chronic condition was approached in very different ways by study participants. Whereas it was easier for some participants to address stressors as a health crisis to be managed in the moment, for others it was important to change the way they viewed their relationship with their illness and, in so doing, change how they managed its effects on their everyday lives and sense of self. Self-management over time has not been well described in the research literature. This study provides evidence of the multiple ways that persons living with chronic illness effectively manage their illness over time.

### Implications for practice

Self-management behavior develops according to different patterns, and therefore, health-care providers might need specific support strategies for various behaviors. For example, peoples’ management of health crises could be better supported by easy access to health-care advice when an event occurs. People who are likely to experience severe episodes or acute events should have access to information beforehand concerning how an event could develop and the possible strategies to address it.

To support everyday life self-management and individual’s transitional processes, health-care providers could partner in ongoing dialogue. In a reflective discussion, the patient and provider could identify the individual’s goals, possible strategies and individualized ways of changing his/her life. Little research has been conducted on how to support transitional processes in people with chronic illnesses, and the topic should be further investigated.

Regarding implications for self-management programs, this study highlights the need for self-management programs to emphasize all aspects of self-management and problems related to life with a chronic condition and not focus on one aspect, for example, secondary prevention or disease knowledge.

## Competing interests

The author has no competing interests.

## Pre-publication history

The pre-publication history for this paper can be accessed here:

http://www.biomedcentral.com/1471-2458/13/452/prepub
